# Hypoxic Effects on Matrix Metalloproteinases’ Expression in the Tumor Microenvironment and Therapeutic Perspectives

**DOI:** 10.3390/ijms242316887

**Published:** 2023-11-28

**Authors:** Georgina Gonzalez-Avila, Bettina Sommer, Edgar Flores-Soto, Arnoldo Aquino-Galvez

**Affiliations:** 1 Laboratorio de Oncología Biomédica, Instituto Nacional de Enfermedades Respiratorias “Ismael Cosío Villegas”, Calzada de Tlalpan 4502, Col. Sección XVI, Tlalpan, Ciudad de México 14080, Mexico; 2 Departamento de Investigación en Hiperreactividad Bronquial, Instituto Nacional de Enfermedades Respiratorias “Ismael Cosío Villegas”, Calzada de Tlalpan 4502, Col. Sección XVI, Tlalpan, Ciudad de México 14080, Mexico; bsommer195@gmail.com; 3 Departamento de Farmacología, Facultad de Medicina, Universidad Nacional Autónoma de México, Coyoacán, Ciudad de México 04510, Mexico; edgarfloressoto@yahoo.com.mx; 4 Laboratorio de Biología Molecular, Departamento de Fibrosis Pulmonar, Instituto Nacional de Enfermedades Respiratorias “Ismael Cosío Villegas”, Calzada de Tlalpan 4502, Col. Sección XVI, Tlalpan, Ciudad de México 14080, Mexico; araquiga@yahoo.com.mx

**Keywords:** cancer treatment, HIFs, hypoxia, matrix metalloproteinases, nanotechnology, tumor microenvironment, transcription factors

## Abstract

The tumor microenvironment (TME) is characterized by an acidic pH and low oxygen concentrations. Hypoxia induces neoplastic cell evasion of the immune surveillance, rapid DNA repair, metabolic reprogramming, and metastasis, mainly as a response to the hypoxic inducible factors (HIFs). Likewise, cancer cells increase matrix metalloproteinases’ (MMPs) expression in response to TME conditions, allowing them to migrate from the primary tumor to different tissues. Since HIFs and MMPs are augmented in the hypoxic TME, it is easy to consider that HIFs participate directly in their expression regulation. However, not all MMPs have a hypoxia response element (HRE)-HIF binding site. Moreover, different transcription factors and signaling pathways activated in hypoxia conditions through HIFs or in a HIF-independent manner participate in MMPs’ transcription. The present review focuses on MMPs’ expression in normal and hypoxic conditions, considering HIFs and a HIF-independent transcription control. In addition, since the hypoxic TME causes resistance to anticancer conventional therapy, treatment approaches using MMPs as a target alone, or in combination with other therapies, are also discussed.

## 1. Introduction

**Figure 1:**
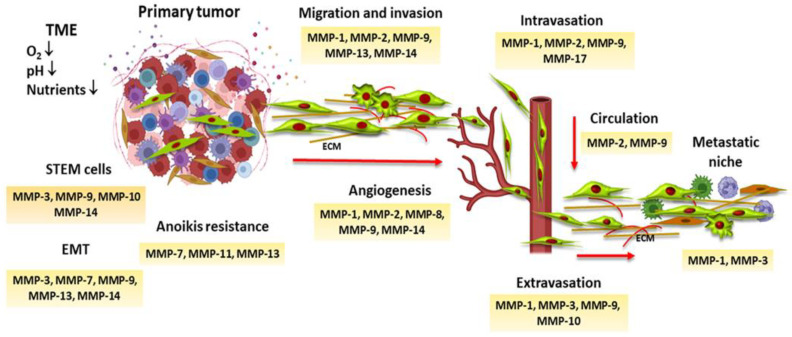
MMPs and the metastatic process. Tumor microenvironment (TME) conditions provoke the expression of MMPs, which defines stem cells’ behaviors, allows neoplastic cells to acquire mesenchymal characteristics through the epithelial–mesenchymal process (EMT), and induces anoikis resistance, which prevents cell death when they detach from the extracellular matrix (ECM) and neighboring cells. MMPs have an important role in angiogenesis, cell migration, invasion, intravasation, and extravasation. They also participate in the formation of the metastatic niche.

Normal tissues are constituted by a heterogeneity of cells and extracellular matrix (ECM) components interacting to preserve tissue homeostasis and organization. To achieve this, cells must coordinate their behaviors, such as cell growth, differentiation, apoptosis, and migration, through complex dynamic mechanisms [1]. Likewise, tissue homeostasis is maintained by cell competition for space, nutrients, and growth factors between cells with higher adaptative skills and cells with a low fitness grade [2]. Moreover, cell competition removes damaged and mutated cells that could evolve into neoplastic cells through mechanisms such as apoptosis, autophagy, senescence, cell differentiation, and epithelial extrusion [2,3]. Cells with a greater adaptative advantage eliminate “loser” cells, maintaining tissue size and homeostasis. However, changes produced in the tissue microenvironment could interfere with the mechanisms that coordinate cell behaviors and provoke genetic and epigenetic alterations in normal and stem cells, transforming them into pre-neoplastic cells with a significant performing capacity to adapt to new tissue conditions and to destroy neighboring wild-type cells in a process called super-competition [4,5,6]. Cell competition may be used during cancer evolution because of the heterogeneity of neoplastic cells that constitute a tumor. The manipulation of cell competition mechanisms allows neoplastic cells to gain space in the surrounding tissue to grow and spread, transforming their microecosystem into their own tumor microenvironment (TME). In this context, cancer cells increase the expression of matrix metalloproteinases (MMPs), which participate in the invasion of the surrounding tissue, as well as in each step of the so-called metastatic cascade that allows tumor cells to colonize tissues distant from the primary tumor (Figure 1) [7].

## 2. The MMPs’ Family

The MMPs’ family includes 28 members in vertebrates, of which only 24 have been identified in humans [7,8]. They are zinc–calcium-ion-dependent endopeptidases grouped according to their protein domains’ arrangement and substrate specificity in gelatinases, collagenases, stromelysins, transmembrane type I MMPs, transmembrane type II MMPs, glycosyl-phosphatidylinositol (GPI)-anchored MMPs, matrilysins, and other MMPs with peculiar characteristics that do not allow them to be included in the previous groups (Table 1) [7].

**Figure 2:**
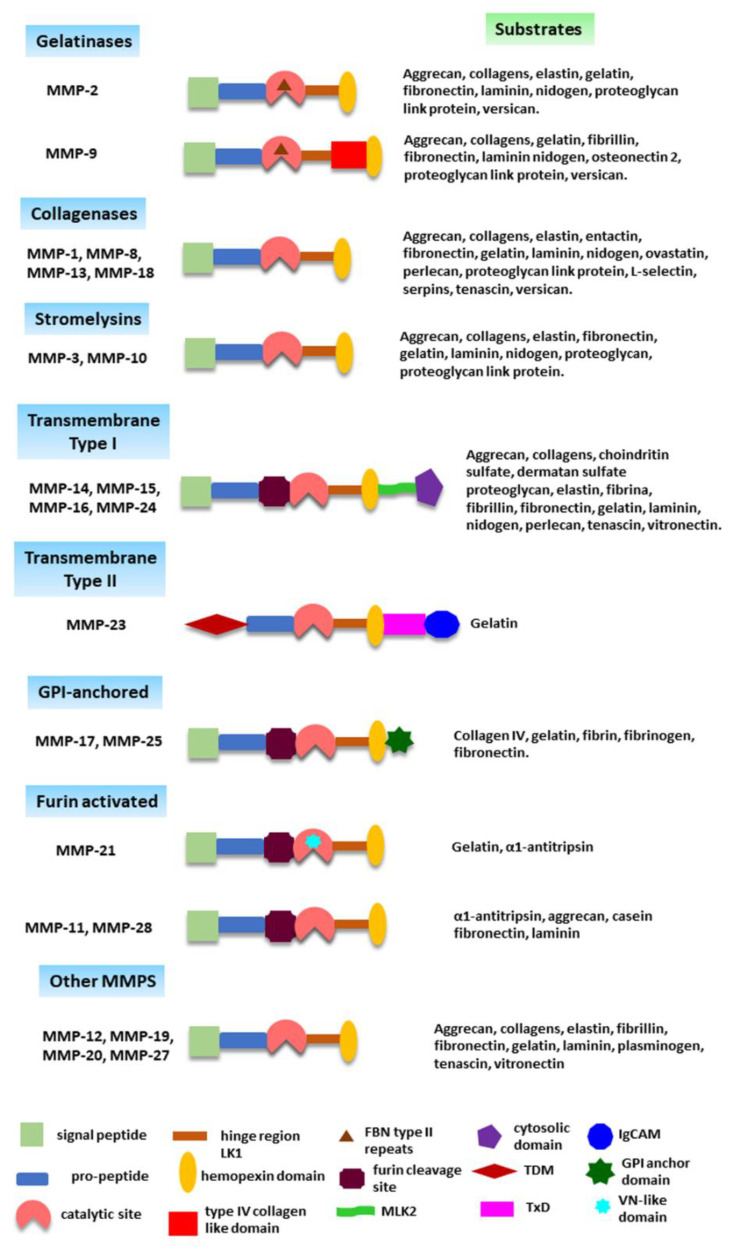
MMPs structure. Most MMPs have a basic structure that contains an N-signal peptide, a pro-peptide, a catalytic motif, a linker 1 (LK1) domain, and a C-hemopexin domain. However, each MMP has other domains that determine their localization and substrate specificity. Abbreviations: GPI = glycosyl-phosphatidylinositol, FBN = fibronectin, IgCAM = immunoglobulin-like cell adhesion molecule MLK2 = membrane linker 2, TDM = transmembrane domain, TxD = toxin-like domain, VN = vitronectin.

The basic structure of most MMPs contains (1) an N-terminal signal peptide; (2) a pro-peptide that includes a cysteine into the PRCGXPD amino acid sequence; (3) a catalytic site containing a zinc ion linked to the cysteine-SH group from the pro-peptide domain (“cysteine switch”), which maintains the MMP in a latent form; (4) a hinge/linker 1(LK1) proline-rich motif; and (5) a C-terminal hemopexin-like region that contributes to the MMP enzymatic activity control, ligand or substrate specificity, and localization [7,9]. Besides this basic structure, other domains are present in MMPs, such as the furin-convertase recognition region localized between the pro-domain and the catalytic site of several MMPs (MMP-11, MMP-14, MMP-15, MMP-16, MMP-17, MMP-23, MMP-24, and MMP-25), or the GPI anchored element situated in the MMP-17 and MMP-25 C-terminal domain. Exceptional is the structure of the MMP-23 that differs greatly from the other MMPs [10]. MMP-23 has no signal peptide but shows a membrane-anchoring transmembrane domain (TDM) at the N-terminal motif that drives the MMP to the Golgi apparatus before its secretion. Moreover, the pro-domain is shorter than in other MMPs and cannot establish a cysteine switch with the catalytic site. In contrast, MMP-23 possesses an RVPGPLAPRRRRY sequence that may bind to the catalytic motif, keeping the enzyme in a latent form. Additionally, MMP-23 has a toxin-like domain (TxD) and an immunoglobulin-like cell adhesion molecule (IgCAM) at the C-terminal region (Figure 2).

## 3. MMPs’ Enzymatic Regulation

MMPs are synthesized as pro-enzymes (latent forms), requiring the disruption of the cysteine switch (except for MMP-23) for their activation, causing a conformational change in the enzyme that allows its binding to the substrate. In this context, modifications in the cysteine switch can occur by the proteolytic disruption of the pro-peptide by the action of plasmin, other MMPs like MMP-3, or trypsin. Likewise, chemicals such as sodium dodecyl sulfate (SDS), mercurial agents, reactive oxygen species (ROS), or glutathione can cause the breaking of the thiol–zinc link, causing a partial activation of the enzyme [11]. Moreover, the link of the substrate with sites outside the MMP catalytic motif (exosites) may provoke an allosteric activation. Then, the MMP total enzymatic activity can be obtained by the autocatalytic elimination of the pro-peptide. In addition, other activation mechanisms have been identified; for example, gelatinases MMP-2 and MMP-9 can be activated by forming the activation complexes pro-MMP-2/TIMP-2/MMP-14 and pro-MMP-9/TIMP-1/MMP-3, respectively [7]. Furthermore, the furin recognition site present in some MMPs can participate in their activation. 

On the other hand, MMPs’ enzymatic activity is mainly regulated by the tissue inhibitors of metalloproteases (TIMPs) [12]. Four TIMPs have been identified: (1) TIMP-1, an inducible 28 kDa glycosylated protein; (2) TIMP-2, a constitutive 21 kDa non-glycosylated protein; (3) TIMP-3, an inducible 24/27 kDa glycosylated protein; and (4) TIMP-4, an inducible 22 kDa non-glycosylated protein. 

TIMPs can be soluble in the extracellular medium or linked to the cell surface, except TIMP-3, which is attached to the ECM or the cell surface. TIMPs’ inhibitory capacity is localized at the N-terminal domain, establishing a non-covalent 1:1 stoichiometry link with the zinc in the catalytic domain. Likewise, the C-terminal motif of TIMP-2 and TIMP-1 participates in the conformation of pro-MMP-2 and pro-MMP-9 activation complexes, respectively [7].

## 4. Regulation of MMPs’ Expression

### 4.1. Transcription Regulation

MMPs’ enzymatic activity regulation is necessary to avoid tissue injury, as seen in fibrosis, inflammation, and cancer metastasis. However, controlling MMPs’ synthesis in neoplastic cells from the primary tumor could prevent their spread to other tissues. Hence, knowing the molecular mechanisms involved in their expression is relevant for cancer treatment. 

MMPs’ production can be conditioned by cytokines, hormones, ultraviolet (UV) irradiation, oncogene products, growth factors, retinoic acids, interleukins, cell–cell interactions, and ECM–cell interactions, as well as changes in the extracellular environment like acidification and hypoxia as occurs in the TME [13,14]. These elements can activate different intracellular signaling pathways and transcription factors such as the mitogen-activated protein kinase (MAPK), nuclear factor-kB (NF-kB), Janus kinase/signal transducer and activator of transcription (JAK/STAT), Wingless and Int-1 (Wnt) signaling, β-catenin/T-cell factor 4/lymphoid enhanced factor (TCF4/LEF), erythroblastosis twenty-six (Ets), runt-related transcription factor-2 (RunX2), and SMAD-3, which are involved in the regulation of MMPs’ gene expression [15,16,17,18]. In this context, several transcription factors’ binding sites have been determined in the promoters of almost all MMPs (Table 2) [19]. 

Additionally, proteins that play a role in some MMPs’ expression have been identified. For instance, Cas interacting zinc finger protein (CIZ) can bind to the consensus sequence (G/C) AAAAA(A) present in MMP-1, MMP-3, and MMP-7, increasing their expression. CIZ, also known as Cas, is a substrate for p130, and it is activated by integrins during cytoskeletal reorganization and cell migration [23]. Furthermore, CIZ forms a complex with the nuclear matrix protein-4 (Nmp4/CIZ) that binds to the MMP-13 parathyroid hormone (PTH), acting synergistically with RunX2 to increase this MMP expression [24].

Likewise, MMP-7 transcription is induced by different stimuli that, in turn, activate NF-κB. For example, the solute carrier family 12 members 5 (SLC12A5), which is overexpressed in bladder urothelial carcinoma cells, regulates MMP-7 expression as well as cell migration and invasion through NF-ĸB-p65 activation [25]. Similarly, sterol-regulatory element binding protein 1 (SREBP1) controls MMP-7 transcription by phosphorylating NF-ĸB-p65 in colon cancer HT29 cells [26]. Moreover, MMP-7 and MMP-10 are upregulated by the phosphatidylinositol-3-kinase/protein kinase B/mammalian target of rapamycin/NF-ĸB (PI3K/Akt/mTOR/NF-ĸB) signaling pathway in gastric epithelial cells infected by *Helicobacter pylori* [20]. These MMPs’ expression is blocked by astaxanthin, decreasing the PI3K/Akt/mTOR/NF-ĸB signaling.

On the other hand, Yan, C. and Boyd, D.D. initially categorized MMPs into three groups according to the structural characteristics of the cis-elements in their promoter regions [16]. This work was enriched by the findings obtained through bioinformatics analysis [19]. The first group is constituted by MMP-1, MMP-3, MMP-7, MMP-9, MMP-10, MMP-12, MMP-13, MMP-19, and MMP-26 in which the promoter zone close to the transcription start point contains a TATA box (−30 pb) and an activator protein-1(AP-1) (−70 pb), with a polyoma enhancer activator protein-3 (PEA-3) binding site upstream. The *trans*-activators that can control their expression are tumor necrosis factor-α (TNFα), platelet-derived growth factor (PDGF), epidermal growth factor (EGF), transforming growth factor-β (TGFβ), vascular endothelial growth factor (VEGF), keratinocyte growth factor (KGF), interleukins (ILs), and basic fibroblast growth factor (bFGF). Likewise, MMP-8, MMP-11, MMP-15, MMP-21, and MMP-27 are part of a second group, whose promoters include a TATA box without a proximal AP-1 zone. It is noteworthy that MMP-11 can be regulated by retinoic acid since three retinoic acid response elements (RAREs), one type direct repeat 1 (DR1)-RARE and two DR2-RARE, are present in its proximal promoter [19]. Interestingly, a retinoic acid response DR1-RARE site was identified in several MMPs through bioinformatic analysis [19]. 

The third group is constituted by MMP-2, MMP-14, MMP-16, MMP-17, MMP-23, MMP-24, MMP-25, and MMP-28. They have many GC box binding sites for specificity protein-1 (Sp1) and Sp3 transcription factors at their proximal promoters and have no TATA box. The synthesis of these MMPs induced by cytokines and growth factors is low, and seemingly, their expression is mainly constitutive [16,19]. However, MMPs’ transcription may depend on other factors. Such is the case for MMP-2 in which an AP-1 site localized at -1394 upstream binds to JunB-FosB heterodimers that induce MMP-2 expression under hypoxic conditions in cardiac fibroblast and myocardial cells, demonstrating that the expression of MMPs can be promoted by the microenvironment around the cells [27].

Likewise, single nucleotide polymorphisms (SNPs) in the promoter regions from MMP-1, MMP-2, MMP-3, MMP-9, and MMP-12 are implicated in the control of their expression [16,18,28]. For example, a G to A transition at ~1576 bp in the MMP-2 promoter affects the interaction of the estrogen receptor α (ERα) and, therefore, the transcription response to estrogen stimulation [16]. Moreover, some MMPs’ promoter polymorphisms have been implicated in the risk of acquiring a disease or in its treatment. Such is the case for the SNP in MMP-9 at −1562 bp, in which the heterozygous genotype CT is associated with the risk for colorectal cancer. In contrast, the heterozygous genotype at −1306 C/T in MMP-2 and the variant genotype GG of MMP-7 −181 A/G reduce the risk of this disease [29]. Likewise, the insertion of a G at −1607 bp at MMP-1 promoter forms the consensus sequence 5′-AAGGAT-3′ instead of 5′-AAGAT-3′, augmenting the Ets transcription factor link and MMP-1 transcription in melanoma cells [30]. Furthermore, SNPs at the promoter regions of MMP-1, MMP-2, MMP-3, and MMP-9 have been identified in head and neck cancers, and therefore, they are considered prognosis markers [28].

Additionally, MMPs’ transcriptional control can be mediated by epigenetic processes. CpG regions susceptible to methylation are present in promoters from MMP-1, MMP-2, MMP-9, MMP-11, MMP-21, MMP-23, MMP-26, MMP-28, and all MT-MMPs. Most of the time, methylation prevents MMPs’ gene transcription by inducing the link of CpG-methyl transcription repressor proteins and consequently blocking the union of transcription factors [31]. For instance, the remotion of the DNA methyltransferases Dnmt-1 and Dnmt-3b induces MMP-3 but not MMP-1 and MMP-2 transcription in colon cancer HTCT116 cells [31,32]. Similarly, CpG hypermethylation at MT1-MMP (MMP-14) and MMP-2 promoter sites decreased the migration of MCF-7 breast cancer cells [31]. Furthermore, acylation of histone H3 and H4 tails by histone acetyltransferase (HAT) relaxes DNA–histone interaction, enabling the access of transcription factors to promoter sites and, therefore, gene expression [33]. On the contrary, hypoacetylation due to histone deacetylases (HDAC) reduces gene transcription. In this context, UV-irradiation of human dermal fibroblast provokes increased p300HAT activity, H3 acetylation, and MMP-1 expression and reduced HDAC enzymatic activity [34]. Likewise, MMP-10 is upregulated in pancreatic ductal adenocarcinoma (PDAC) cells resistant to chemotherapy, in which gemcitabine increased H3 acetylation [35].

### 4.2. Post-Transcriptional Regulation

MMPs’ protein expression can be regulated at the post-transcriptional level by microRNAs (miRNAs) and RNA-binding proteins that interact with target mRNAs through the 3′-untranslated regions (UTRs). The miRNAs are single-stranded non-coding RNAs with 17 to 26 nucleotides that bind to complementary sequences at the UTRs of RNA transcripts, blocking their translation or destabilizing them by decapping, shortening the poly-A-tail, or allowing 5′ to 3′ exonucleolytic decay [36]. Several miRNAs that interfere with MMPs’ translation have been identified. For instance, miRNA-539 suppresses MMP-8 expression and proliferation, invasion, and migration of osteosarcoma cells, and miRNA-125, which targets MMP-13, interferes with the proliferation and spread of cutaneous squamous cell carcinoma cells [37,38]. Likewise, there is evidence that miRNAs involved in MMPs’ regulation are downregulated in cancer. Such is the case of miRNA-16 levels that are decreased in advanced stages of non-small cell lung cancer (NSCLC) [39]. This miRNA suppresses MMP-9 expression and metastatic characteristics of A549 lung cancer cells. Similarly, low miRNA-183 and high MMP-9 levels were observed in tissue samples of cervical cancer tissue [40]. Most miRNAs participate in the regulation of the synthesis of a single MMP; however, miRNA-29b regulates MMP-2 synthesis in prostate cancer cells and MMP-9 expression in osteosarcoma, while miRNA-34a controls MMP-9 and MMP-14 production in tongue squamous cell carcinoma [41,42,43]. For more detailed information about miRNAs and their interaction with MMPs in cancer progression, refer to Grzywa, T.M., et al. [36].

Likewise, another mechanism to control transcript turnover and translation is the linking of RNA binding proteins (RNABPs) to adenine uracil (AU)-rich elements (AREs) at the 3′-UTR [44]. Twenty ARE-binding proteins (AUBPs) have been identified. Among them is the Hu protein family, constituted by HuR, HuB, HuC, and HuD, which participate in mRNA stability. In contrast, proteins involved in mRNA degradation are AU-rich element RNA binding protein-1 (AUF-1), butyrate-regulated factor-1 (BRF1), tristetraprolin (TTP), and KH splicing regulatory protein (KSRP).

MMPs’ transcripts can be modulated by mRNA stabilization. For instance, MMP-9 mRNA has four ARE sites at the 3′UTR, but only HuR binds to ARE1, ARE2, and ARE4, stabilizing MMP-9 transcript in rat renal mesangial cells [45]. Furthermore, nitric oxide decreases HuR and increases AUF-1 expression with the subsequent MMP-9 mRNA degradation [45,46]. 

Additionally, stabilization of the MMP-9 transcript by HuR and HuB has been observed after rat hippocampal neuron activation by pentylenetetrazole in which HuR is bound to ARE1 and ARE4 while HuB is attached to ARE1 [47]. Likewise, MMP-2 and MMP-9 transcripts’ half-life was increased by 6 to 8 h and 6.5 to 9 h, respectively, when mouse smooth muscle cells (SMCs) were transfected with a HuR plasmid [48]. Moreover, angiotensin II increases HuR linking to MMP-2 and MMP-9 mRNA and HuR expression through the NF-κB pathway. These effects are inhibited by SMCs pre-treatment with melatonin. On the contrary, HuR can have different post-transcriptional effects on other MMPs. For instance, MMP-13 transcript concentrations increase in hypoxic conditions while MMP-3 levels are augmented in both normoxia and hypoxia in HuR knockdown rat nucleus pulposus cells [49]. Likewise, the interaction of HuR with MMP-1 can be disturbed by the circular RNA cirDLC1 in hepatocellular carcinoma cells (HCC), causing an increase in MMP-1 transcript degradation [50]. Furthermore, cirDLC1 is considered a good prognosis marker since its upregulation not only decreases MMP-1 expression but also reduces hepatoma cells’ proliferation, invasion, and migration abilities. 

Another protein that can bind to mRNA 3′-UTR regions participating in transcript stabilization is nucleolin (NCL). For example, NCL increases MMP-2 transcript half-life and cell invasiveness properties in human bladder T24T cells [51]. Additionally, MMP-7 cleaves NCL at the N-terminal site (Asp255), and the truncated form (TNCL) enhances cancer cells’ proliferation, invasion, and migration capacities. Moreover, TNCL binds more effectively to the MMP-9 3′-UTR zone than the full NCL, reducing MMP-9 transcript degradation in H1299 and A549 lung cancer cell lines [52]. Moreover, MMP-7 knockdown reduced MMP-9 mRNA expression, protein levels, and cells’ metastatic behavior. These findings demonstrate the existence of the MMP-7/TNCL/MMP-9 axis in cancer cells.

## 5. The Hypoxic TME

The TME comprises different cell types, extracellular structural and non-structural components, and a complex and changing metabolic environment that directs tumor cells to improve their fitness capacity to evolve [53]. In the primary tumor, neoplastic cells augment their proliferation rates, enhancing energy requirements and oxygen consumption, therefore diminishing oxygen and nutrient concentrations. Moreover, as the tumor grows, the distance between cancer cells and blood vessels increases (>200 µm), significantly obstructing oxygen diffusion [54]. On the other hand, new vessels generated by angiogenesis are tortuous and un-organized, without the pericyte cover and gaps between the tumor endothelial cells (TECs), allowing blood leaking and inducing alterations in blood flow with the production of blood clots, promoting further the decrease in oxygen distribution [55]. Additionally, factors such as carcinogenic agents, drugs, microbiota dysbiosis, and acidification of the microenvironment can interfere with endothelial cell functions [56]. Similarly, few red blood cells (RBCs) or hypoxic RBCs may cause poor oxygen diffusion into the tumor mass [55]. Moreover, the remodeling of the ECM by cancer and stromal cells causes vascular compression and blood supply diminution [56]. 

Likewise, tumors are constituted by areas showing different degrees of hypoxia. Some zones have mild hypoxia (<10 mmHg); in contrast, others display severe hypoxia (<1 mmHg) or even necrosis (anoxia), while others present acute or chronic hypoxia [57]. Furthermore, periods of low oxygen concentrations followed by re-oxygenation episodes were observed, although with different patterns, among distinct tumor types [58]. This intermittent hypoxia is involved in the acquisition of neoplastic cells’ metastatic characteristics and probably in the increase in MMPs’ expression and enzymatic activity. 

## 6. The MMPs in the Acidic TME

Hypoxia provokes a switch between the oxidative phosphorylation and the anaerobic glycolysis (Warburg effect) in tumor cells, therefore increasing the production of lactic acid and inducing the acidification of the TME (pH from 6.4 to 7.0) [59]. Acidic pH induces changes in the neoplastic cells’ morphology showing increased membrane filopodia and invadopodia. In addition, acidic pH enhances the transferring of exosomes containing proteolytic enzymes, such as MMPs and cathepsin B, to the membrane protrusions, preparing cells to migrate through the ECM. Moreover, the plasma membrane N^+^/H^+^ exchanger 1 (NHEI) contributes to the peri-invadopodia acidic pH, which increases MMP-2, MMP-9, and cathepsin B enzymatic activity and secretion [60]. Additionally, cathepsin B participates in the pro-MMP-2 activation as an alternative to the pro-MMP-2/MMP-14/TIMP-2 activation complex, also located at the invadopodia [61]. 

Likewise, acidic pH increases the formation of membrane invaginations called caveolae, structurally formed by cavins and caveolins. Caveolae protect cells from mechanical stress and participate in signal transduction and endocytosis. They have receptors involved in MMPs and cathepsin B activation, such as urokinase plasminogen receptor (uPAR), S100A10, and enolase-1 (ENO-1) [14,59]. Furthermore, caveolae contribute to the peri-cellular acidic pH since they contain NHEI and Nav1.5, which augment H^+^ efflux. The role of ENO-1 is interesting since, besides participating in regulating the Warburg effect, it also functions as a receptor for urokinase plasminogen activator (uPA), which transforms plasminogen into plasmin. Plasmin, in turn, releases cytokines and growth factors from de ECM and activates several pro-MMPs such as MMP-1, MMP-2 (associated with MMP-14), MMP-3, MMP-9, MMP-12, MMP-13, and MMP-14 [62].

## 7. The Hypoxic Inducible Factors (HIFs)

Cells can sense oxygen level reduction through several molecules present in their plasmatic membrane and inside the cells, which, in turn, activate different pathways in order to survive. One of these mechanisms involves the hypoxic inducible factors (HIFs) [63]. HIFs are transcription factors constituted by an alpha chain (HIF-1α, HIF-2α, also known as EPAS1, or HIF-3α), whose expression and stabilization depend on oxygen concentrations. HIFs contain an aryl hydrocarbon receptor nuclear translocator (ARNT) or ARNT2 subunit known as HIF-1β and HIF-2β, respectively [64]. The beta subunits are independent of the oxygen concentrations; however, ARNT is overexpressed in hypoxic conditions in some cells.

Additionally, when cells are in a normoxic microenvironment, the HIFα subunits located in the cytoplasm are hydroxylated in a specific proline residue: HIF-1α on Pro-402 and Pro-564, HIF-2α on Pro-405 and Pro-531, and HIF-3α on Pro-492 located at the oxygen-dependent degradation (ODD) motif [63]. The hydroxylation is carried out by the prolyl-4-hydroxylases (PHDs) 1-3. These enzymes belong to the α-ketoglutarate dioxygenases, which can monitor oxygen levels and require oxygen, α-ketoglutarate, and Fe^2+^ for their activity. Once HIFs-α chains are hydroxylated, they bind to the von Hippel–Lindau protein (pVHL), preparing them for ubiquitination and subsequent proteasomal degradation. Mutations in the VHL gene inhibit HIFs-α breakdown, inducing HIFs’ pathway and the transcription of genes involved in cancer progression [65]. 

In the hypoxic TME, the PHDs cannot hydroxylate HIFs-α chains, leading to their stabilization and translocation to the nucleus, where they dimerize with the β-subunits. The dimers form a complex with the coactivator cAMP-response-element binding protein (CREB)-binding protein (CBP)/p300, which links to the hypoxia response element (HRE). The HRE sequence 5′G/ACGTG3′ is situated near the promoters or enhancers of the target genes. HIF-1α and HIF2α bind to this HRE core sequence, but HIF-1α does it proximally and HIF-2α distally, to the transcription starting site [66]. Moreover, a different HRE core sequence 5′-(G/A)(T/C)(A/G)(C/G)G(T/A) for HIF-2α has been identified in cells exposed to hypoxia. 

HIFs/CBP/p300 complex formation can be prevented by the factor inhibiting HIF-1 (FIH-1), which hydroxylates asparagine residues (HIF-1α on Asn-803 residue and HIF-2α on Asn851) at the C-terminal transactivation region in the presence of oxygen [63]. 

Likewise, HIF-1α expression can be regulated by the PI3K/AKT/mTOR signaling pathway, fumarate hydratase (FH), succinate dehydrogenase (SDH), phosphatase and tensin homolog (PTEN), protein kinase A (PKA), and Ra4/MAPK pathway under hypoxic conditions or independently of oxygen concentrations due to mutations of any of these components, [67,68,69,70]. 

**Figure 3:**
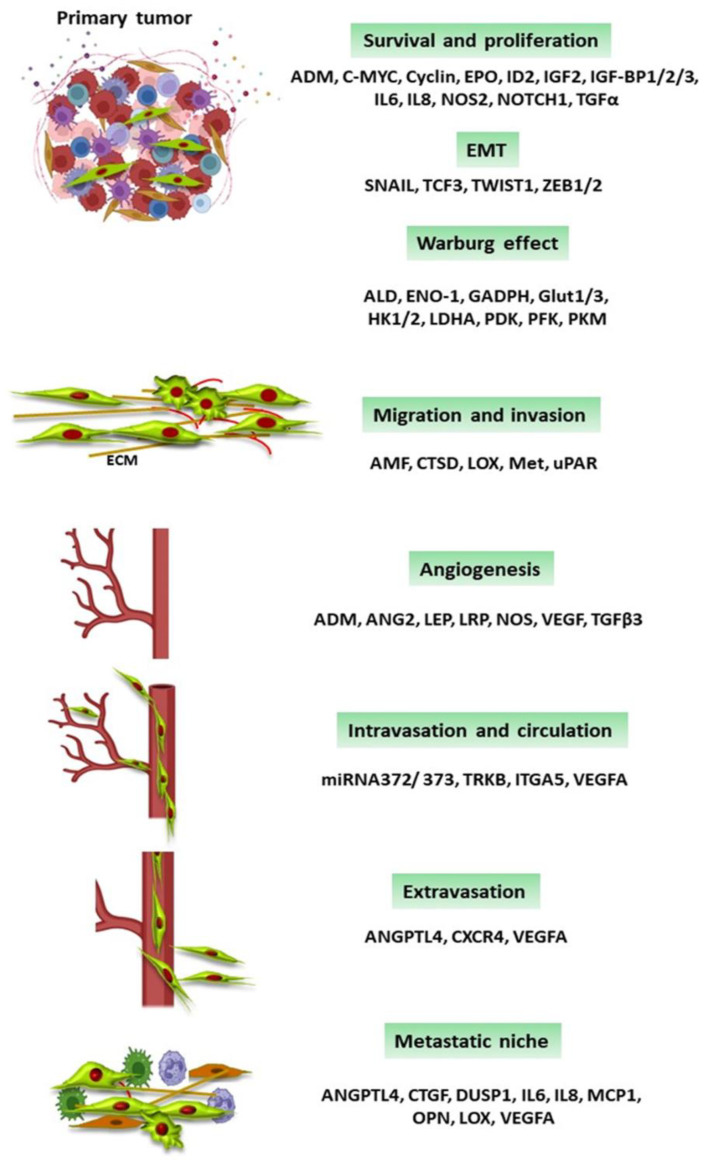
HIFs’ target genes in cancer metastasis. Low oxygen concentrations induce the expression of the hypoxia-inducible factors (HIFs). HIFs are transcription factors and their target genes are involved in all the steps of the metastatic cascade. Abbreviations: ADM = adrenomedullin; ALD = aldolase; AMF = autocrine motility factor; ANG2 = angiotensin converting enzyme 2; Angiopoietin-like 4; c-MYC = myelocytomatosis virus oncogene cellular homolog; CTSD = cathepsin D; CXCR4 = C-X-C chemokine receptor type 4; CTGF = connective tissue growth factor; DUSP1 = dual specificity protein phosphatase 1; ECM = extracellular matrix; ENO-1 = enolase-1; EPO = erythropoietin; GADPH = glycelaldehido-3-phosphate dehydrogenase; GLUT1/3 = glucose transporter1/3; HK1/2= hexokinase 1/2; ID2 = DNA-binding protein inhibitor; IGF2 = insulin-like growth factor 2; IGF-BP1/2/3 = IGF-factor-binding protein 1/2/3; IL-6 = interleukina-6, IL-8 = interleukina-8, ITGA5 = integrin alpha 5; LDHA = lactate dehydrogenase; LEP = leptin; LOX = lysyl oxidase; LRP1 = LDL-receptor-related protein1; MCP1 = monocyte chemoattractant protein-1; NOS = nitric oxide synthase; OPN = osteopontin; PDK = pyruvate dehydrogenase kinase; PFK = phosphofructokinase; PKM = pyruvate kinase M; TCF3 = transcription factor 3; TGF-α = transforming growth factor α; TGF-β3 = transforming growth factor-β3; uPAR = urokinase plasminogen receptor; TrkB = tropomyosin receptor kinase B; VEGF = vascular endothelial growth factor; VEGFA = vascular endothelial growth factor A; ZEB 1/2 = zinc finger E-box-binding homeobox 1/2.

HIFs are involved in the transcription of different genes that participate in glucose transport, glycolysis, neoplastic cell survival, proliferation, angiogenesis, migration and invasion, and the preparation of the metastatic niche. Some of these genes are shown in Figure 3.

## 8. MMPs Expression and HIFs Hypoxic Conditions

Since MMPs and HIFs are overexpressed under hypoxic conditions in the TME, it has been considered that HIFs may be involved in regulating MMPs’ transcription. Various studies have been conducted to confirm this assumption, but a direct or indirect correlation between MMPs’ expression and HIFs’ regulation has been found only for some MMPs.

### 8.1. MMP-1

MMPs’ expression under hypoxic conditions can be regulated by different factors, including HIFs. For instance, in vitro experiments showed that HIF-1α, MMP-1, and MMP-3 expression increased when human bone marrow mesenchymal stem cells (bmMSCs) were cultured with 2% oxygen for 4 h. Transfection of these cells with HIF-1α siRNA decreased MMP-1 and MMP-3 mRNA levels [71]. Likewise, HIF-1α and MMP-1 were upregulated in lung adenocarcinoma cells exposed to hypoxia (2% oxygen) for 40 h [72]. Moreover, MMP-1 synthesis decreased with the use of HIF-1α siRNA. Interestingly, the induction of MMP-1 can be mediated by Est-1, a HIF-1α target gene. Therefore, HIF-1 participates indirectly in MMP-1 transcription regulation [21,73]. Likewise, chondrosarcoma cells exposed to 2% oxygen for 48 h showed a C-X-C chemokine receptor type 4 (CXCR4) overexpression, which augmented MMP-1 transcription [74]. CXCR4 is the receptor for the stromal-cell-derived factor 1 (SDF1); therefore, stimulation with this chemokine in hypoxic conditions further increases MMP-1 expression. Furthermore, SDF1 induces extracellular signal-related kinase (ERK) phosphorylation. In this context, ERK inhibitor U0126 or the CXCR4 inhibitor AMD3100 reduces MMP-1 synthesis and cell invasion capacity; thus, the CXCR4 effect on MMP-1 expression is through the ERK signaling pathway. In addition, CXCR4 transcription is regulated by HIF-1α, which contributes indirectly to MMP-1 expression. Furthermore, ERK participates in HIF-1α activation by Ser-641 and Ser-643 phosphorylation [75]. CXCR4 can upregulate other MMPs’ expression, such as MMP-2, MMP-8, MMP-9, and MMP-13, which HIF-1α can indirectly control. Similarly, gastric cancer 58As9 cells exposed to 1% oxygen for 12, 24, and 48 h showed an increase in MMP-1 expression, which decreases in HIF-1α knockdown cells and with the use of the HIF-1α inhibitor YC-1 [76].

### 8.2. MMP-2

MMP-2 expression has also been studied under hypoxic conditions. For example, the exposition of mouse colon cancer ES cells to 1% oxygen for 24 h showed an upregulation of HIF-1α and MMP-2 that decreased with the use of HIF-1α siRNA or in knockout HIF-1α^−/−^ cells cultured in normoxic conditions. [77]. Similarly, HIF-1α siRNA decreases MMP-2 expression in esophageal cancer Eca109 cells in a hypoxic model with CoCl_2_ at 6, 12, and 24 h [78]. The overexpression of HIF-1α in this in vitro model, besides MMP-2 upregulation, increases Snail and reduces E-cadherin synthesis. Snail is a HIF-1α target gene that regulates the epithelial–mesenchymal transition (EMT) process and E-cadherin expression. Hence, MMP-2 and E-cadherin expression can be regulated by HIF-1α through Snail, favoring invasion and metastasis of esophageal cancer cells under hypoxic conditions. 

In addition, patients with colorectal cancer (CRC) showed an association between increased levels of MMP-2, HIF-1α, and zinc finger protein384 (ZFP384) in tissue samples, and poor survival outcomes [79]. Moreover, these proteins are overexpressed under hypoxic conditions (0.5% oxygen) in in vitro experiments. HIF-1α knockdown cells decreased ZFP384 expression, and direct mutagenesis analysis revealed two HIF-1α cis-binding sites in the ZFP384 promoter, demonstrating that it is a HIF-1α target gene. Furthermore, ZFP384 links directly to a specific site in the MMP-2 promoter, controlling its expression. Therefore, the HIF-1α effect on CRC invasion capacity is due to ZFP384 transcription increasing MMP-2 expression.

On the other hand, the exposition of glioma U87MG cells to 1% oxygen demonstrated an increase in HIF-1α and MMP-2 transcription with a rise in cell invasion and migration capacities [80]. Moreover, the use of HIF-1α siRNA blocks HIF-1α and MMP-2 expression. Additionally, MMP-9 synthesis was not affected by hypoxia or HIF-1α siRNA in this in vitro model. Interestingly, hypoxia enhances MMP-2 and MMP-9 enzymatic activity, which decreases in HIF-1α siRNA transfected cells.

### 8.3. MMP-9

MMP-9 transcription under hypoxic conditions differs from that of MMP-2. For example, MMP-9 expression can be regulated by arylamine N-acetyltransferase 1 (NAT1) through HIF-1α in a cell in vitro model [81]. Interestingly, breast cancer MDA-MB-231 and T47D cells that had NAT1 gen deleted showed increased MMP-9 expression. Furthermore, histone 3 (H3), associated with the SET and MYND-domain containing 3 (SMYD3) at the MMP-9 promoter (−325 to −309 bp), was more acetylated in NAT1 knockdown cells, facilitating MMP-9 transcription. Contrastingly, HIF-1α transcription was not different in NAT1 knockdown and NAT1-expressing cells. However, HIF-1α siRNA reduces MMP-9 transcription in NAT1 knockdown cells. Therefore, NAT1 may interfere with chromatin structure, blocking HIF-1α or a HIF-1α target protein to interact with the MMP-9 promoter site.

Likewise, coactivators such as CBP/p300 participate in the regulation of MMP-9 expression. CBP/p300 has histone acetyltransferase activity to relax the chromatin structure, allowing transcription factor binding [82]. Moreover, CBP/p300, together with coactivator-associated arginine methyltransferase 1 (CARM1), glucocorticoid-receptor-interacting protein 1 (GRIP1), and p300/CBP-associated factor (PCAF), forms a transcriptional complex involved in MMP-9 expression.

Interestingly, the MMP-9 luciferase reporter gene and chromatin immunoprecipitation (ChIP) assays carried out on breast cancer MDA-MB-231 cells under hypoxic conditions, showed that HIF-1 forms a complex with p300 that increases MMP-9 promoter activity, including H3 and H4 acetylation [83]. This activity was induced by the Ras-GTP pathway, which participates in HIF-1α stabilization. 

Similarly, the parathyroid hormone-related protein (PTHrP) is involved in the expression of MMP-9, VEGF, and HIF-1α through the PI3K/AKT and ERK1/2 signaling pathways in colon cancer Caco-2 and HCT-116 cells [84]. Moreover, tumor-conditioned media from cells exposed to the PTHrP stimulates endothelial HMEC cells’ migration and vessels’ tube formation in an in vitro angiogenesis model in which MMP-9 and VEGF play an essential role. In this regard, MMP-9 and VEGF transcription could be induced by HIF-1α, whose stabilization is regulated by the PI3K/AKT and ERK1/2 pathways; however, the use of AKT, PI3K, and ERK1/2 inhibitors blocks MMP-9 and VEGF transcription, suggesting that their expression regulation may be carried out directly by PI3K/AKT and ERK1/2.

### 8.4. MMP-13

Another MMP whose expression regulation has been examined in hypoxic conditions is MMP-13. In an ovarian cancer cell in vitro model, an increase in MMP-13 and HIF-1α mRNA levels under hypoxia was observed, showing a Pearson correlation of 0.8813 (*p* < 0.001) [85]. In addition, MMP-13 transcription and protein levels decreased in cells transfected with HIF-1α siRNA under hypoxia. Moreover, ChIP assays demonstrated an HRE site in the MMP-13 promoter for HIF-1α binding in human nasopharyngeal carcinoma (NPC) CNE2 cells [86]. Interestingly, HIF-1α and MMP-13 were localized in cancer cells and exosomes when cells were exposed to hypoxia. Hypoxic exosomes can be taken up by neighboring cells in which these molecules induce the EMT and migration processes. Furthermore, exposure of human umbilical vein endothelial cells (HUVEC) to these hypoxic exosomes stimulated endothelial cells’ proliferation and vessels’ tube formation as part of the angiogenesis phase.

Likewise, hypoxia induces the expression of the transcription coactivator eyes absent 3 (EYA3) regulated by HIF-1α and HIF-2α in an in vitro CRC model [87]. Then, EYA3 forms the transcriptional complex EYA3-sine oculis homeobox homolog 5 (SIX5)-p300, which, in turn, regulates MMP-3, MMP-7, MMP-8, MMP-21, and MMP-26 expression. Each member of this complex has a specific function: SIX5 can bind to target genes’ promoters, p300 acetylates histones to relax the chromatin structure, and EYA3 acts as a SIX5 coactivator for MMPs’ transcription, inducing cancer progression. Moreover, the EYA3 inhibitor benzarone blocks the transcription complex formation, MMPs, VEGFD, and EGFR expression, as well as tumor growth, and neoplastic cell invasion capacity in mouse tumor xenograft and in vitro cell models.

### 8.5. MMP-14

Studies performed in an in vitro mice bone-marrow-derived mesenchymal stromal cell (MSC) model subjected to hypoxic conditions (1% oxygen for 48 h) revealed that HIF-1α upregulated MMP-14 (MT1-MMP) [88]. Moreover, HIF-1α siRNA inhibits MMP-14 transcription and MSCs’ migration capacity. Besides its proteolytic activity and participation in pro-MMP-2 activation, MMP-14 with HIF-1α can regulate 3BP2 adaptor protein expression, favoring MSCs’ migration in hypoxic environments. 

Interesting is the relation between HIF-1α and MMP-14. For instance, HIF-1α expression can be regulated by the early growth response protein-1 (EGR-1) transcription factor stimulated by hypoxia [89]. Moreover, experiments conducted with rat endothelial cells cultured in a three-dimensional (3D) collagen matrix, demonstrated that EGR-1 can also control MMP-14 transcription in endothelial cells when the ECM is modified during angiogenesis since this MMP has in its promoter a binding site (−288 to −275 pb) for EGR-1 [90]. Therefore, it is difficult to determine whether, in hypoxic conditions, MMP-14 expression is a consequence of HIF-1α, EGR-1, or both.

In addition, FIH-1 interferes with the HIF-1α/CBP/p300 transcription complex formation, contributing to HIF-1α degradation. However, the munc-18-1 interacting protein 3 (Mint3) N-terminal domain interacts with FIH-1, suppressing HIF-1α hydroxylation and elimination [91]. Furthermore, MMP-14 binds to FIH-1, favoring Mint3-FIH-1 interaction leading to HIF-1α activation. This effect is only possible in those regions where MMP-14 is located since its cytoplasmic domain binds to FIH-1, facilitating the interaction with Mint3. Considering all this information, it seems that a feedback loop exists between HIF-1α and MMP-14 since HIF-1α promotes the transcription of this MMP, and MMP-14 favors the activation of HIF-1α. Further studies must elucidate the relationship between both molecules and whether this happens under hypoxic conditions in all neoplastic cells.

Likewise, in vitro experiments demonstrated that MMP-14 and HIF-2α are overexpressed in metastatic renal cell carcinoma (RCC) cells, which synthesized a truncated non-functional pVHL protein [92]. Moreover, MMP-14 and HIF-2α downregulation was observed in transfected RCC cells with pVHL, while an increase in MMP-14 transcription occurred in VHL^−/−^ and HIF-2α^+/+^ cells. This finding showed that MMP-14 expression depends on HIF-2α. Additionally, a HIF-2α binding site was identified at the MMP-14 proximal promotor (−125 bp). However, HIF-2α requires Sp1 for the regulation of MMP-14 transcription. The binding of both HIF-2α and Sp1 probably enhances the expression of MMP-14; thus, further research is required.

Unlike HIF-1α, HIF-2α is only expressed in some cell types, such as the clear cell subtype of RCC (ccRCC), the most prevalent type of renal cancer [93]. In this regard, ccRCC cells have mutations in the VHL gene and, in 40% of the cases, in the HIF-1α gene with a HIF-2α overexpression due to decreased degradation. In addition, ccRCC is the leading cause of death in von Hippel–Lindau disease patients, whose aggressiveness could be due to the increase in MMP-14 expression among other molecules involved in cancer progression. Therefore, inhibitors are being developed that prevent the expression of proteins regulated by HIF-2α, including MMP-14, VEGFA, glucose transporter 1 (GLUT1), and cyclin D1. 

### 8.6. MMP-15 and MMP-17

MMP-15 (MT2-MMP) is upregulated by HIF-1α under hypoxia conditions (1% oxygen) in pancreatic cancer PANC-1 cells since its transcription is inhibited by HIF-1α siRNA or the YC-1 HIFs’ inhibitor [94]. In addition, an HRE site was identified in the MMP-15 promoter site (−369 to −252 bp) to which HIF-1α binds directly. 

Similarly, MMP-17 (MT4-MMP) and HIF-1α were overexpressed in hypopharyngeal squamous cell carcinoma FADU, tongue squamous cell carcinoma SAS, and OECM-1 cells when they were incubated under hypoxia conditions (1% oxygen) for 18 h [95]. The hypoxia effects were blocked in HIF-1α siRNA transfected cells. Moreover, results from transactivation assays demonstrated that MMP-17 has an HRE site (−399 to −395 bp) in its proximal promoter, which could permit the direct interaction of HIF-1α. Furthermore, an E-box site (−457 to 4462 bp) was identified in the MMP-17 proximal promoter; this characteristic allows its transcription regulation by SLUG, a target gene of HIF-1α. Removing the E-box site decreases MMP-17 expression in hypoxic conditions. Therefore, MMP-17 transcription under hypoxia could be regulated directly by HIF-1α and through SLUG in these types of cancer. 

## 9. The HIF-1α/IL-6/JAK/STAT/MMPs Axis

HIF-1α regulates IL-6 and IL-8 transcription, increasing their expression [96]. Once IL-6 is expressed, it attaches to its receptor-α, allowing gp130 activation and the subsequent JAK2 phosphorylation (JAK2-p). JAK2-p, in turn, favors STAT-3 activation, which binds to the MMP-9 promoter under chronic hypoxia, increasing basement membrane (BM) degradation and microvascular proliferation [97]. Moreover, IL-6 can be secreted by inflammatory cells and cancer-associated fibroblasts (CAFs) in the hypoxic TME, favoring the activation of IL-6/JAK/STAT, EMT promotion, and gastric cancer cells’ invasion [98]. Therefore, the hypoxic TME may facilitate the activation of the HIF-1α/IL-6/JAK/STAT/MMPs axis in which different MMPs can participate because they have a STAT site in their promoters (Table 2). Such is the case of MMP-1, MMP-2, MMP-3, and MMP-9, in which transcription is stimulated by IL-6 [99]. Interestingly, STAT-1 can bind to STAT binding elements (SBEs) localized in MMP-1 and MMP-3 proximal promoters in complex with AP-1 (Fos/c-Jun) components favoring their transcription.

Hence, IL-6 could be a cancer progression inducer upregulated by hypoxia since the activation of this signaling pathway in hypoxic conditions by HIF-1α is associated with several steps of the metastatic cascade in which the MMPs are involved.

## 10. HIF-1α, Toll-like Receptors, and MMPs

Toll-like receptors (TLRs) are transmembrane proteins that activate the adaptive and innate immune response [100]. Until now, 13 TLRs have been identified in mammalians with different cellular localization: TLR3, TLR7, TLR8, and TLR9 are found in the endosomal membrane, while TLR1, TRL2, TLR5, and TLR6 are present in the cell membrane. Interestingly, TLR4 is present in both. In the TME, TLRs are expressed in immune cells such as lymphocytes T, lymphocytes B, and macrophages. However, TLRs are also localized in the neoplastic cells, playing a role in cell proliferation and invasion. In this regard, TLRs are involved in MMPs’ expression. For instance, TLR9, MMP-2, MMP-9, and MMP-13 expression is upregulated, while TIMP-3 is downregulated under hypoxic conditions (1% oxygen for 24 h) in glioma U373MG and D54MG cells. TLR9 siRNA blocks cells’ invasion and these MMPs’ transcription [101]. 

Likewise, the TLR9 agonist unmethylated oligonucleotide CpG, which is recognized as a bacterial DNA by TLR9, increases MMP-13 expression and activity, reduces TIMP-3 synthesis and the invasion capacity of breast MDA-MB-231 cancer cells [102]. Although these studies were not performed under hypoxic conditions, they indicate a correlation between TLR9 and the MMPs’ expression in neoplastic cells. 

Additionally, TLR9 induces MMPs’ expression by activating NF-κB and AP-1. For example, esophageal TE10 cancer cells incubated with the TRL9 agonist CpG increase NF-κB expression, which in turn upregulates MMP-2, MMP-7, and cyclooxygenase-2 (COX-2) transcription as well cell migration [103]. This effect is blocked by chloroquine (CQ), a TRL9 inhibitor. Moreover, agonist activation of TRL9 can stimulate the STAT-3 signaling pathway in mouse B16 melanoma cells, which, in turn, can activate MMPs’ transcription, such as MMP-9 [22]. 

In addition, an association of HIF-1α with TLR9 expression has been established in which HIF-1α siRNA decreases TLR9 transcripts in glioblastoma multiforme (GBM) cells treated with insulin-like growth factor 1 (IGF-1) under normoxic conditions [104]. These findings indicate that TLR9 could be a HIF-1α target gene; therefore, HIF-1α regulates MMPs’ transcription through TLR9 in hypoxic environments. Thus, more studies are required.

Likewise, TLR4 is upregulated by HIF-1α in hypoxic conditions in macrophages, as well as TLR2 and TLR6 in murine bone-marrow-derived dendritic cells (BMDCs) exposed to 2% oxygen for 24 h [105,106]. Moreover, HIF-1α binding sites have been identified in TLR2 and TLR6 promoters by ChIP assays in human microvascular endothelial HMEC-1 cells [105]. Additionally, TLR2 expression is induced in glioma-associated macrophages (GAM) by glioma cells provoking the transcription of MMP-9 and MMP-14, and cancer growth and spread [107]. Furthermore, the TLR2 agonist Pam3CSK4 augments MMP-9 and MMP-14 expression while the O-Vanillin suppresses this effect in in vitro experimental models. Interestingly, GAM are type M2 macrophages, which contribute to cancer progression. In this context, type M1 macrophages have an immune-suppressive function in the tumor normoxic zones, but in the hypoxic TME, macrophages polarize to M2 cells by the effect of IL-4 and IL-13 [14,108]. In addition, TLR2 transcription is regulated by versican released from glioma cells. Versican is a HIF-1α target gene [109]. Therefore, TLR2 expression from GAM cells could be regulated directly and indirectly by HIF-1α, increasing MMP-9 and MMP-14 transcription.

## 11. HIF-Independent MMPs’ Expression Regulation

Hypoxia regulates HIFs’ expression and activity through different pathways such as the TLR4/NF-ĸB, IL6/JAK/STAT3, PI3K/AKT/mTOR, MAPK, Notch, sonic Hedgehog (sHH), and Wnt/βcatenin signaling pathways [56,110]. Likewise, hypoxia stimulates the NADPH oxidase (NOX) system, which is involved in the activation of oncogenes, like RAS, EGFR, and Src, and the inhibition of oncogenic suppressor genes (p53, tuberous sclerosis complex 2 (TSC2), and PTEN), through the production of ROS [111]. Moreover, ROS generated by NOX participates in HIF-1α stabilization, which regulates NOX1, NOX2, and NOX4 expression, demonstrating the existence of feedback between NOX and HIF-1α [112]. Additionally, p22phox, NOX1, and NOX2 induce HIF-2α transcription in VHL-deficient renal cell carcinoma. Interestingly, the pathways activated by hypoxia involved in HIF expression can also directly activate MMPs’ transcription in a HIF-independent manner. Such is the case of the sHH-Gli pathway, which, when inhibited with cyclopamine, decreases breast cancer MDA-MB-231 cells’ invasion and migration, and MMP-2 and MMP-9 concentrations [113]. Moreover, rhSHH upregulates MMP-2 and MMP-9 expression and protein levels, while cyclopamine downregulates mRNA and protein concentrations in glioma U251 cells [114]. In addition, rhSHH increases Akt phosphorylation and MMP-2 and MMP-9 synthesis, while the PI3K inhibitor Ly294002 blocks shSHH effects. These findings indicate that the sHH-Gli signaling participates in MMP-2 and MMP-9 expression through the PI3K/Akt pathway. Likewise, Gli1 siRNA decreases MMP-2 and MMP-9 expression and enzymatic activity and the invasion and migration capacity of the hepatoma SMMC-7721 and SK-Hep1 cells in in vitro experiments [115]. This effect could be due to the direct interaction of the transcription factor Gli1 or through the PI3K/Akt pathway as occurs in breast cancer cells. Additionally, MMP-11 has been identified as a target gene of Gli1 with a Spearman correlation of 0.295, which is low compared with other target genes such as PTCH1 (r = 0.47) or FOXS1 (r = 0.372) in embryonal rhabdomyosarcoma Rh36 and medulloblastoma Daoy cells [116]. Furthermore, LEF1 was also identified as a Gli1 target gene but with a low correlation (r = 0.172) in Rh36 and Daoy cells. Some, MMPs, including MMP-3, MMP-7, and MMP-9, have a binding site for this transcription factor (Table 2). 

On the other hand, besides stimulation of HIFs’ expression, other transcription factors are upregulated in hypoxic environments. For example, treatment with the TX-402 inhibitor of HIF-1α expression downregulates the transcription of HIF-1α target genes GLUT-1 and erythropoietin but not MMP-7, MMP-14, or Ets-1 when hepatocellular carcinoma HepG2 and Hep3B were cultured in hypoxic conditions (1% oxygen) [117]. Moreover, in Hep3B cells transfected cells with the HIF-1α dominant negative vector (pHIF-1αDN), HIF-1α is downregulated while MMP-7, MMP-14, and Ets-1 mRNA levels did not decrease compared to non-transfected cells in hypoxic conditions. In this regard, MMP-7 and MMP-14 have an Ets-1 binding site, and therefore, their expression is controlled independently of HIF-1α (Table 2). Likewise, other transcription factors like EGR1, AP-1, and NF-ĸB, which are involved in MMPs’ expression, are directly regulated by hypoxia [118].

## 12. Therapy Resistance under a Hypoxic Environment

Cancer treatments include chemotherapy, target therapy, immunotherapy, and radiotherapy, which lose effectiveness in a hypoxic environment [119]. For instance, co-cultivation of CAFs with CRC cells in hypoxic conditions favors chemotherapy resistance to a combination of 5-Fluorouracil (5-FU) and oxaliplatin, cancer cell stemness, and dedifferentiation. CAFs secrete TGFβ2, which, together with HIF-1α from the CRC cells, induced GLI2 expression without the canonical Hedgehog pathway participation [120]. GLI2, in turn, induces the expression of B-cell lymphoma extra-large (Bxcl-xL) and X-linker inhibitor of apoptosis protein (XIAP), which have anti-apoptotic properties. Likewise, some MMPs can induce chemoresistance. Such is the case of MMP-1, whose downregulation lowers thymidylate synthase and dihydropyrimidine dehydrogenase levels, increasing 5-FU activity in in vitro experiments performed with nasopharyngeal cancer cells [121]. 

In addition, hypoxia increases the fibroblast growth factor receptor-1 (FGFR1) expression, which induces osimertinib (AZD9291) resistance via the MAPK pathway [122]. Osimertinib is a third-generation drug that belongs to the epidermal growth factor receptor (EGFR) tyrosine kinase inhibitors (TKIs) used in advanced lung adenocarcinoma with EGFR mutations. Moreover, an NSCLC cell and tissue bioinformatic analysis demonstrated that the MMP-1 gene was overexpressed in erlotinib (a TKI) resistance NSCLC cells through its association with COPS5, which is involved in drug resistance [123]. Furthermore, the MMP-1 epigenetic-regulated transcription factors HOXA9 and PBXI were also upregulated in erlotinib-resistant cells. Similarly, other MMPs, such as MMP-2, MMP-7, MMP-9, and MMP-14, have been implicated in chemotherapy and target therapy resistance [124].

On the other hand, the hypoxic TME generates an immunosuppressive environment that leads to the chimeric antigen receptor (CAR) T-cells and immune checkpoint inhibitors (ICPIs) resistance [125]. Similarly, radiotherapy is one of the most used cancer treatments causing tumor cell death by direct damage of DNA due to the generation of abasic sites, DNA-single strand breaks, DNA-double strand breaks, DNA base damage, and complex DNA damage, or ROS production, which directly produce DNA lesions [126]. However, neoplastic hypoxic cells are radio-resistant since they can control the DNA damage response, ROS regulatory systems, glycolysis, and cell cycle [127].

## 13. MMPs’ in Cancer Treatment

To overcome cancer treatment resistance due to hypoxia, several strategies are in development, such as tissue re-oxygenation, target HIFs (HIF inhibitors) or molecules that are part of the HIF pathway, pro-drugs activated in an acidic pH, and target molecules that play a role in the metastatic process under hypoxia conditions [128]. Among them, MMPs have been considered therapeutic targets to control cancer progression. MMP inhibitor therapy involves different perspectives: (1) direct inhibition of their enzymatic activity, (2) gene expression control, (3) protein synthesis blockage, and (4) suppression of pro-enzymes’ activation. 

### 13.1. MMPs’ Synthetic and Natural Inhibitors

Greater importance has been given to the design of synthetic MMP inhibitors (sMMPIs) that interfere with the MMPs’ enzymatic activity. sMMPIs include thiirane-based slow inhibitors, peptidomimetic inhibitors, non-peptidomimetic inhibitors, chemically modified tetracyclines, and off-target inhibitors (Table 3) [129,130,131,132]. These inhibitors have been included in clinical protocols to establish safety doses and determine possible side effects [132]. However, side effects such as musculoskeletal syndrome characterized by myalgias, arthralgias, and tendinitis, as well as gastrointestinal disorders, caused their disuse as therapeutic agents. In this context, immunotherapy using monoclonal antibodies against MMPs’ catalytic motif acting as a competitive inhibitor or targeting other enzyme sites that lead to an allosteric inhibition has been used to avoid side effects and increase specificity [133]. For instance, GS-5745 (Andecaliximab) and DX-2400, which inhibit MMP-9 and MMP-14, respectively.

Similarly, natural products that can inhibit MMPs’ activity have been considered to avoid side effects (Table 3). For example, genistein (soya isoflavone) suppresses MMP-2, MMP-9, MMP-14, MMP-15, and MMP-16 activity [135]. Moreover, genistein also blocks NF-ĸB activation and decreases Akt protein concentrations, interfering with MMPs’ expression [139].

Additionally, phenolic natural compounds can have MMPI characteristics like mangiferin that inhibit MMP-9, isosylibin, which targets MMP-13, and isoliquiritigenin, which can block MMP-1, MMP-2, MMP-3, MMP-7, MMP-8, MMP-9, and MMP-13 enzymatic activity [138]. These phenolic substances can inhibit human osteosarcoma U-2OS cells migration capacity and tumor growth and metastatic lesions in a U-2OS tumor-bearing mice model. 

Likewise, the polyphenol epigallocatechin gallate (EGCG), a component of green tea, suppresses the activity and binding of transcription factors and signaling pathways such as NF-ĸB, AP-1, JNK, ERK1/2, STAT-3, Sp-1, and PI3K/AKT involved in MMPs’ expression, through its ROS regulation attributes [137]. Moreover, EGCG can interact directly with MMP-2 and MMP-9, inhibiting their enzymatic activity. Furthermore, EGCG linkage to MMP-14 blocks pro-MMP-2 activation. Similarly, baicalein, a flavonoid derived from the root of *Scutellaria baicalensis*, can also inhibit the function of transcription factors like NF-ĸB, Sp-1, STAT-3, and the PI3K/AKT/mTOR, MAPK and ROS signaling pathways, among others interfering with MMP-2 and MMP-9 expression [134]. In this regard, baicalein downregulates MMP-2 expression by suppressing the p38 MAPK/NF-ĸB pathway in ovarian cancer cells [145]. Likewise, ginseng has been employed in different inflammatory diseases and is considered an anti-inflammatory and anticancer agent because of its effects on the NF-ĸB pathway. In this context, current studies reveal that the ginsenoside Rh1 from *Panax ginseng* interferes with STAT-3 phosphorylation and disrupts the nuclear translocation of NF-ĸB subunits that, in turn, downregulates MMP-2 and MMP-9 expression in breast cancer MDA-MB-231 cells [136].

### 13.2. MicroRNAs

Likewise, transfecting cells with miRNA that downregulated MMPs’ protein synthesis can be used for therapeutic purposes (Table 3). For example, miRNA-146a downregulates MMP-1, uPA, and uPAR in breast cancer MDA-MB-435-LvBr2 cells, and miRNA-143 downregulates MMP-13 in osteosarcoma cells, decreasing neoplastic cells’ migration and invasion potential [140]. Other miRNAs that can be used to target MMPs are included in Table 3.

Interestingly, miRNA-21 decreases TIMP-3 and reversion-inducing cysteine-rich protein with Kazal motifs (RECK) mRNA levels, both MMPs’ enzymatic activity inhibitors [146]. Moreover, cells transfected with anti-miRNA-21 oligonucleotide showed decreased MMP-2 activity, a rise in RECK and TIMP-3, and reduced glioma cells’ migration.

Similarly, besides miRNAs, the use of MMPs’ siRNA can be considered a therapeutic approach in cancer.

### 13.3. Nanoparticles

Nanomaterials can participate in the inhibition of MMPs’ enzymatic activity, transcription, and protein synthesis (Table 3). For instance, low-size (5 to 10 nm) gold nanoparticles (AuNPs) inhibit MMP-2 and MMP-9 transcription, cell proliferation, and invasion capacity of thyroid cancer SW579 cells [147]. Additionally, a combination of carbon quantum dots (CQDs) with cuprous oxide (Cu_2_O) NPs has been employed to target ovarian cancer progression [141]. CQDs have low cytotoxicity, biocompatibility, photoluminescence, and water solubility, while Cu_2_O NPs have pro-apoptotic properties and reduce metastatic lesions. Cu_2_O/CQDs can interfere with MMP-2, MMP-9, and VEGFR2 expression, reducing the invasion and migration of ovarian cancer SKOV3 cells. Furthermore, Cu_2_O/CQDs interfere with HUVEC’s new vessel formation. 

Likewise, NPs can target a signaling pathway involved in MMPs’ expression. Such is the case of gallic acid functionalized with AuNPs (Ga-AuNPs), which inhibits MMP-9 but not MMP-2 expression [142]. Ga-AuNPs block the EGF/EGFR pathway, which activates Akt/NF-ĸB and ERK/c-Jun and promotes p300 stabilization necessary for MMP-9 expression. Similarly, the metallofullerenol Gd@C82(OH)22 NPs can block MMP-2 and MMP-9 synthesis and activity, significantly affecting MMP-9 [143]. Moreover, Gd@C82(OH)22 NPs bind close to the S1 exosite, provoking an allosteric inhibition of MMP-9 activity. Additionally, resveratrol NPs suppress MMP-2 and increase TIMP-2 expression, decreasing renal cancer ACHN and A498 cells’ migration and invasion capacities [144]. Furthermore, resveratrol NPs’ effects on MMP-2 synthesis are due to a reduction in p-ERK1/ERK2 expression. 

Other nanomaterials that can interfere with MMPs’ expression and enzymatic activity are revised by Shi Y. et al. [132].

## 14. Conclusions and Future Directions

Hypoxia provokes changes in the TME to which neoplastic cells adapt to survive and evolve, acquiring metastatic characteristics that allow them to detach from the primary tumor and migrate through the systemic circulation to form a metastatic colony. Likewise, MMPs’ synthesis increase is a characteristic of the neoplastic cells in the hypoxic TME together with the increase in HIFs expression; hence, it has been considered that MMPs’ transcription would be regulated by HIFs under hypoxic conditions. However, as we point out in the present review, not all MMPs have HARE sequences for HIFs binding. Moreover, their regulation can be carried out through other molecules, which are HIFs’ targets. In addition, transcription factors and signaling pathways involved in MMPs’ expression can be addressed by hypoxia in a HIF-independent manner. 

On the other hand, since MMPs play an important role in cancer progression, new strategies that target MMPs’ expression and enzymatic activity must be developed. In this context, MMPs and HIFs suppressor agents in combination with conventional cancer treatment, can be included in a nanoplatform to avoid toxic side effects and improve the delivery to the target tumor [119]. In this regard, the use of HIFs’ inhibitors can modify TME conditions, enhancing other therapies’ effectivity, including therapy based on MMPs’ inhibition. Likewise, nanosystems could take oxygen or oxygen transporters like perfluorochemical emulsions, oxygen generators (manganese dioxide (MnO_2_) and catalase), and hemoglobin-based oxygen carriers to reverse hypoxic conditions [148]. Nanocarriers can be combined with other techniques to improve tumor oxygenation, such as hyperthermia, normobaric and hyperbaric hyperoxia respiration, and antiangiogenic drugs to normalize tumor vasculature. These new therapies, together with conventional treatment, may control cancer progression. 

In addition, nanoplatforms can be designed with theranostic purposes to localize the tumor, directing at the same time several treatments to the neoplastic tissue and follow-up treatment response. In this regard, MMPs can be employed for diagnosing and monitoring cancer progression using nanobiosensors and imaging nanotechniques that depend on MMPs’ enzymatic activity [149]. They can also direct and activate nanosystems in the tumor tissue and can be part of the therapy included in the nanocarrier.

In summary, the regulation mechanisms of MMPs’ expression are complex under hypoxic conditions and depend on the MMP and neoplastic cell type.

Hopefully, the information presented in this review helps to find more effective therapies to interfere with MMPs’ expression and, thus, prevent cancer progression. However, more questions arose during the development of the present review, which led to the conclusion that there is still much to discover.

## Figures and Tables

**Table 1:** MMPs’ Classification.

Group	MMP
Gelatinases	MMP-2 (gelatinase A) MMP-9 (gelatinase B)
Collagenases	MMP-1 (interstitial collagenase 1) MMP-8 (collagenase 2/neutrophil collagenase) MMP-13 (collagenase 3) MMP-18 (collagenase 4)
Stromelysins	MMP-3 (stromelysin 1) MMP-10 (stromelysin 2) MMP-11 (stromelysin 3)
Transmembrane Type I	MMP-14 (MT1-MMP) MMP-15 (MT2-MMP) MMP-16 (MT3-MMP) MMP-24 (MT5-MMP)
Transmembrane Type II	MMP-23 (CA-MMP)
GPI anchored	MMP-17 (MMT4-MMP) MMP-25 (MMT6-MMP)
Matrilysins	MMP-7 (matrilysin 1) MMP-26 (matrilysin 2)
Other MMPs	MMP-12 (macrophage elastase) MMP-19 (RASI-1) MMP-20 (enamelysin) MMP-21 MMP-27 MMP-28 (epilysin)

This table was constructed based on the information reported in reference [7]. GPI = glycosyl-phosphate inositol, MMP = matrix metalloproteinase, MT-MMP = membrane type MMP.

**Table 2:** Transcription Factors Involved in MMPs’ Expression Regulation.

MMP	AP-1	AP-2	DR1-RARE	DR3-RARE	ETs	NF-ĸB	RunX2	Sp1/Sp3	STAT	TCF/LEF
**MMP-1**	+	---	+	---	+	+	+	---	+	+
**MMP-2**	---	---	+	---	---	+	---	+	+	---
**MMP-3**	+	---	---	---	+	---	---	---	+	+
**MMP-7**	+	---	+	---	+	+	+	---	+	+
**MMP-8**	+	+	+	---	+	+	+	---	---	+
**MMP-9**	+	---	+	---	---	+	---	+	+	+
**MMP-10**	+	---	---	---	+	+	---	---	+	+
**MMP-11**	+	---	+	---	+	+	---	---	---	---
**MMP-12**	+	---	---	---	+	---	---	---	+	---
**MMP-13**	+	---	+	---	+	+	+	---	+	---
**MMP-14**	---	---	+	---	+	+	---	+	---	+
**MMP-15**	---	+	---	---	+	+	---	---	+	---
**MMP-16**	---	+	---	---	+	---	---	+	---	---
**MMP-17**	+	+	+	+	---	+	+	+	+	---
**MMP-19**	+	+	+	---	---	+	---	---	---	+
**MMP-20**	+	+	---	---	---	---	---	---	---	---
**MMP-21**	---	+	+	+	+	---	+	---	---	+
**MMP-23a**	+	+	+	---	---	+	---	+	+	+
**MMP-24**	---	+	---	---	+	+	---	+	---	---
**MMP-25**	---	+	---	---	+	+	---	+	---	---
**MMP-26**	+	---	+	+	+	+	+	---	---	+
**MMP-27**	+	+	---	---	+	---	---	---	---	---
**MMP-28**	+	+	---	---	---	---	---	+	+	+

This table was constructed based on the findings reported in references: [15,16,17,19,20,21,22]. AP-1 = activator protein-1, DR1-RARE = direct repeat 1-retinoic acid response element, Ets = erythroblastosis twenty-six, MMP = matrix metalloproteinase, NF-ĸB = nuclear factor ĸB, RunX2 = runt-related transcription factor-2, Sp1 = specificity protein-1, STAT = signal transducer and activator of transcription, TCF/LEF = T-cell factor/ lymphoid enhancer factor.

**Table 3:** Different Types of MMPIs Used in Cancer Treatment.

Therapy Strategy		MMPs Specificity	Reference
sMMPIs			
Thiirane-based slow inhibitors	ND-322	MMP-2, MMP-14	[130]
	SB-3CT	MMP-2, MMP-9	[131]
Chemical modified tetracyclines	Metastat (COL-3)	MMP-1, MMP-2, MMP-8, MMP-9, MMP-13	[129,130,131,132,133]
	Doxycycline (Periostat)	MMP-1, MMP-2, MMP-8, MMP-9	[129,130,131,132,133]
	Minocycline (Minocin)	MMP-2. MMP-9	[129,130,131,132,133]
Peptidomimetic inhibitors	Batismastat (BB-94)	MMP-1, MMP-2, MMP-3, MMP-7, MMP-9	[129,130,131,132,133]
	Marimastat (BB-2516)	MMP-1, MM-2, MMP-7, MMP-9, MMP-14	[129,130,131,132,133]
Non-peptidomimetic inhibitors	CGS-27023A	MMP-1, MMP-2, MMP-3, MMP-8, MMP-9	[129,130,131,132,133]
	Prinomastat (AG3340)	MMP-2, MMP-3, MMP-7, MMP-9, MMP-13, MMP-14	[129,130,131,132,133]
	Rebimastat	MMP-1, MMP-2, MMP-8, MMP-14	[129,130,131,132,133]
	Tanomastat	MMP-2, MMP-3, MMP-7, MMP-9, MMP-13, MMP-14	[129,130,131,132,133]
Sulfonamide derivatives	Disulfiram	MMP-2, MMP-9	[129,130,131,132,133]
	S3304	MMP-2, MMP-9	[129,130,131,132,133]
Off-target MMPI	Letrozole (non-steroidal hormone)	MMP-2, MMP-9	[129,130,131,132,133]
	Zoledronic acid (bisphosphonate)	MMP-2, MMP-9, MMP-14, MMP-15	[129,130,131,132,133]
**Immunotherapy** **(monoclonal antibodies)**			
	AB0041, AB0046	MMP-9	[133]
	DX-2400	MMP-14	[133]
	GS-5745	MMP-9	[133]
**Natural inhibitors**			
	Baicalein	MMP-2, MMP-9	[134]
	Genistein (soya isoflavone)	MMP-2, MMP-9, MMP-14, MMP-15, MMP-16	[135]
	Ginesoide Rh1 (*Panax ginseng*)	MMP-2, MMP-9	[136]
	Neovastat AE941 (shark cartilage)	MMP-1, MMP-2, MMP-7, MMP-9, MMP-12, MMP-13	[133]
**Phenolic compounds**	Epigallocatechin gallate (EGCG)	MMP-2, MMP-9	[137]
	Isoliquiritigenin	MMP-1, MMP-2, MMP-3, MMP-7, MMP-8, MMP-9, MMP-13	[138]
	Isosylibin	MMP-13	[138]
	Mangiferin	MMP-9	[138]
**MicroRNAs**			
	miRNA-16	MMP-9	[39]
	miRNA-29b	MMP-2, MMP-9	[41,43]
	miRNA-34a	MMP-9, MMP-14	[42]
	miRNA-125	MMP-13	[37]
	miRNA-143	MMP-2, MMP-9, MMP-13	[139]
	miRNA-146a	MMP-1	[140]
	miRNA-183	MMP-9	[40]
	miRNA-539	MMP-8	[38]
**Nanoparticles**	AuNPs	MMP-1, MMP-2, MMP-8, MMP-9	[132]
	AgNPs	MMP-2, MMP-3, MMP-9	[132]
	Cu_2_O/CQDs	MMP-2, MMP-9	[141]
	Fe_3_O_4_@PO	MMP-2	[132]
	Fullurenols	MMP-1, MMP-3, MMP-13	[132]
	GaAuNPs	MMP-9	[142]
	Gd@C_82_(OH)_22_	MMP-2, MMP-9	[143]
	PtNPs	MMP-1, MMP-2, MMP-8, MMP-9	[132]
	Resveratrol NPs	MMP-23	[144]
	SeHAN-NPs	MMP-9	[132]
	SeNPs	MMP-2	[132]
	WO_3_ NPs	MMP-7	[132]
	ZnO NPs	MMP-9	[132]

MMPs = matrix metalloproteinases, MMPIs = matrix metalloproteinases inhibitors, sMMPIs = synthetic matrix metalloproteinases inhibitors, NPs = nanoparticles, SeHAN = Selenium-substituted hydroxiapate.

## Data Availability

All data included in this review were found in the original articles cited in the text.
